# Patients with reduced heart rate response to adenosine infusion have low myocardial flow reserve in ^13^N-ammonia PET studies

**DOI:** 10.1007/s10554-015-0654-6

**Published:** 2015-04-07

**Authors:** Takeshi Tomiyama, Shin-ichiro Kumita, Keiichi Ishihara, Masaya Suda, Minoru Sakurai, Kenta Hakozaki, Hidenobu Hashimoto, Naoto Takahashi, Hitoshi Takano, Yasuhiro Kobayashi, Tomonari Kiriyama, Yoshimitsu Fukushima, Wataru Shimizu

**Affiliations:** Department of Radiology, Nippon Medical School, 1-1-5 Sendagi, Bunkyo-ku, Tokyo, 113-8603 Japan; Clinical Imaging Center for Healthcare, Nippon Medical School, Tokyo, Japan; Department of Cardiovascular Medicine, Nippon Medical School, Tokyo, Japan

**Keywords:** Heart rate response, Adenosine, Myocardial blood flow (MBF), Myocardial flow reserve (MFR), ^13^N-ammonia, Positron emission tomography (PET)

## Abstract

To assess the effect of adenosine infusion by evaluating the relationship between heart rate (HR) response to adenosine and myocardial flow reserve (MFR) of remote regions supplied by normal coronary arteries in ^13^N-ammonia PET. Thirty-one consecutive subjects (20 known coronary artery disease patients, 4 chronic heart failure patients, and 7 normal volunteers) except cases having 3-vessel disease underwent rest and adenosine stress ^13^N-ammonia myocardial perfusion PET. Semi-quantitative, quantitative, and gated analyses were performed. Subjects were divided into two groups with regard to HR response to adenosine. Twenty-two subjects had normal HR response (peak/rest HR > 1.20), while reduced HR response (≤1.20) was observed in nine subjects. There were no differences in rest myocardial blood flow (MBF) of remote regions between the groups. Subjects with reduced HR response had significantly lower stress MBF and MFR of remote regions than those with normal HR response (stress MBF: 1.559 ± 0.517 vs. 2.279 ± 0.530, *p* = 0.004, MFR: 1.59 ± 0.36 vs. 2.35 ± 0.53, *p* = 0.001). There were no significant differences between the groups by means of semi-quantitative scoring. Rest and stress ejection fraction (EF) in the reduced HR response group was lower than that in the normal HR response group. In a multiple stepwise regression analysis, HR ratio, dyslipidemia, and Brinkman index were identified as predictors of the change in MFR of remote regions. Subjects with reduced HR response to adenosine had lower stress MBF and MFR of remote regions and lower EF. Moreover, HR response was one of the predictors of the change in MFR of remote regions.

## Introduction

In adenosine stress myocardial perfusion single photon emission computed tomography (SPECT) imaging, reduced blood flow is estimated according to the degree of hyperemia between normal and stenosed coronary arteries. Adenosine induces approximately a four-fold increase in coronary blood flow [1]. Adenosine generally yields an increase in heart rate (HR) and a decrease in blood pressure (BP). Reduced HR response to adenosine or dipyridamole was reported to relation with abnormal perfusion, low ejection fraction (EF), and cardiac autonomic neuropathy [2–5]. Moreover, patients undergoing adenosine stress perfusion SPECT with high rest HR and low peak/rest HR ratio have an increased risk of cardiac death [6, 7].

^13^N-ammonia myocardial perfusion positron emission tomography (PET) is generally performed under pharmacologic stress condition because the half-life of ^13^N-ammonia is only about 10 min. Myocardial perfusion PET with ^13^N-ammonia can evaluate the absolute rest and stress myocardial blood flow (MBF) and myocardial flow reserve (MFR) [8, 9]. Reduced MFR occurs due to coronary stenosis, and microvascular and vascular endothelial dysfunctions [10–13]. Stress MBF and MFR should be assessed under the proper adenosine stress test. The aim of this study was to validate heterogeneity of hemodynamic response to adenosine, and to assess the association between hemodynamic response and MFR as well as diagnostic performance of myocardial ischemia in ^13^N-ammonia PET studies.

## Materials and methods

### Study population

Thirty-one consecutive subjects (22 males, 9 females; mean age ± SD, 66.3 ± 11.8 year) underwent rest and adenosine stress ^13^N-ammonia myocardial perfusion PET. Twenty subjects had known coronary stenosis: 1-vessel stenosis in 8 patients and 2-vessel stenosis in 12 patients. Eleven subjects had no known coronary artery stenosis: seven subjects were normal volunteers, and four subjects underwent PET studies to assess chronic heart failure. Normal volunteers were in good health and had never undergone coronary computed tomography (CT) angiography or coronary angiography (CAG), but some of the subjects had hypertension, dyslipidemia, or diabetes mellitus. Cases with 3-vessel coronary artery disease were excluded because MBF and MFR of all coronary territories were influenced by coronary artery stenosis. Coronary stenosis was defined as >50 % diameter stenosis shown by CAG. Exclusion criteria with regard to the adenosine test were asthma, pacemaker rhythm, second or third degree atrioventricular block, or sick sinus syndrome. Written informed consent was obtained from all subjects. This study was approved by the local ethics committee and was performed in accordance with the ethical standards of the Declaration of Helsinki.

### Data acquisition and adenosine stress protocol

All subjects had fasted for >6 h and had refrained from caffeine-containing beverages and food, smoking, and medications for 24 h prior to imaging. PET scan was performed on a GEMINI TF-16 (Philips Medical Systems), which is a hybrid time-of-flight (TOF)-PET/CT scanner [14]. PET acquisition was performed in the three-dimensional list mode. The scatter correction was a TOF single scatter simulation algorithm (TOF-SSS), while the attenuation correction was based on rest and stress cardiac CT scans. At rest, approximately 370 MBq of ^13^N-ammonia was injected into a peripheral vein of all subjects, followed by a 30 mL saline flush. Dynamic and gated acquisition was initiated just before injection, and was extended for 20 min. Adenosine stress test was performed more than 50 min (5 half-lives) after rest protocol. During this time, ^13^N activity at rest had physically decayed to about 3 % of its initial activity. Adenosine was infused at 120 µg/kg/min for 6 min, according to Japanese clinical conventions. ^13^N-ammonia and adenosine were infused through the peripheral veins of both the right and left arms (2-route method) to prevent suspending adenosine infusion during administration of ^13^N-ammonia. No patient exercised during adenosine stress test. ^13^N-ammonia of the same dose (370 MBq) was injected at the end of the 3 min of administration. HR, BP, and a 12-lead electrocardiogram (ECG) were obtained every minute during and after adenosine infusion, with continuous ECG monitoring. Twenty-five dynamic frames were reconstructed (10 × 5, 10 × 6, 1 × 10, 2 × 30 s, 1 × 1, and 1 × 6 min, for a total of 10 min). The reconstruction parameters for TOF-ordered subset expectation maximization (OSEM) were set at three iterations and 33 subsets as optimized for the clinical application.

During stress tests, hemodynamic response to adenosine was recorded. Peak HR was defined as the highest HR during the observation period. HR ratio was calculated by dividing the peak HR by the baseline HR. A reduced HR response to adenosine was considered if the HR ratio was 1.20 or less, according to previous reports [5–7]. Participants were divided into two groups according to HR ratio: normal and reduced HR response groups. Minimum BP was defined as the lowest mean BP during the observation period. Significant ST depression was defined as ≥1 mm of either horizontal or down sloping or ≥1.5 mm of up sloping; ST-segment depression was measured 80 ms after the J point.

### Image interpretation

Images were re-sliced in the short-axis, as well as in the vertical and horizontal long-axis orientations. Regional ^13^N-ammonia accumulation was assessed using the American Heart Association (AHA) 17-segment model, and the semi-quantitative scoring system of defect severity and extent [15]. A 5-point scale scoring method was used, as follows: 0, no defect; 1, mildly reduced; 2, moderately reduced; 3, severely reduced; and 4, absent of activity. Image interpretation was performed by two experienced readers. Discordance of scoring was classified by consensus. For each image, a summed rest score (SRS) and a summed stress score (SSS) were calculated by adding the segment scores at rest and stress, respectively. A summed differential score (SDS) was calculated by subtracting the SRS from the SSS.

Quantitative analysis was performed using PMOD software package (version 3.4, PMOD Technologies Ltd., Zurich, Switzerland). Regions of interest were semi-automatically placed on the left ventricular myocardium, and the left and right ventricular blood pools in each slice. The time-activity curves of myocardial and blood pool, generated from the dynamic frames, were fitted with the tracer kinetic model (DeGrado 1-compartment model) [16]. Only the first 4 min of data were used for the curve fitting. The estimated rest and stress MBF were expressed in each segment and territory, while the MFR was calculated by dividing the stress MBF by the rest MBF. MBF and MFR in remote regions supplied by normal coronary arteries were used for statistical analyses: global values for normal coronary disease, mean values of normal 2-territory for 1-vessel disease, and values of normal 1-territory for 2-vessel disease. Rest MBF values modified by rate-pressure product (mean values of 31 subjects: 8800) were also calculated. Rest and post-stress end-diastolic volume (EDV), end-systolic volume (ESV), and EF were derived from each image using cardioREPO software (FUJIFILM RI Pharma Co. Ltd,) [17].

### Statistics

All statistical analyses were performed using SPSS version 19 for Windows (SPSS Inc., Chicago, IL). Continuous variables are expressed as mean ± SD. Categorical variables were compared between normal and reduced HR response groups using Chi square tests. The Wilcoxon rank sum test was used to compare nonparametric variables between the groups. A Pearson’s product moment correlation coefficient and a standard linear regression analysis were also used to describe the correlation between the HR ratio and quantitative values. Multiple stepwise regression analysis was used to determine predictors as appropriate. MFR of remote regions was used as dependent variable, and gender, age, body mass index (BMI), hypertension, diabetes mellitus, dyslipidemia, estimated glomerular filtration rate (eGFR), Brinkman index, the number of coronary artery stenosis, symptom, HR ratio (as numerical values), ΔBP, symptoms and ST changes during adenosine infusion, rest EDV, rest ESV, and rest EF were the independent variables. A *p* value <0.05 was deemed statistically significant. Moreover, sensitivity, specificity, positive predictive value (PPV), and negative predictive value (NPV) for myocardial ischemia in normal and reduced HR response groups were calculated. Myocardial ischemia was defined if the visual stress score was higher than the rest one for each territory. A gold standard of ischemia was >50 % stenosis on CAG.

## Results

Twenty-two subjects had normal HR response, while reduced HR response was observed in nine subjects; 5 of 20 coronary artery disease patients, 3 of 4 chronic heart failure patients, and 1 of 7 normal volunteers had reduced HR response. Baseline characteristics of the two groups are shown in Table 1. Subjects with reduced HR response frequently had a smoking habit (*p* = 0.015) and tended to take β-blocker (*p* = 0.074). There were no differences between the groups in gender, age, BMI, history of hypertension, diabetes mellitus, dyslipidemia, kidney dysfunction, the number of abnormal coronary arteries, and symptoms.

**Table 1:** Baseline characteristics according to normal or reduced heart rate ratio

	Normal HRR (n = 22)	Reduced HRR (n = 9)	*p* value
Gender (male)	14 (63.6 %)	8 (88.9 %)	0.160
Age (y/o)	65.5 ± 11.8	68.2 ± 11.5	0.571
Body mass index (kg/m^2^)	23.7 ± 3.9	22.6 ± 4.2	0.572
Hypertension	15 (68.2 %)	7 (77.8 %)	0.593
Diabetes mellitus	9 (40.9 %)	3 (33.3 %)	0.694
Dyslipidemia	14 (63.6 %)	6 (66.7 %)	0.873
Hemodialysis	1 (4.5 %)	2 (22.2 %)	0.131
eGFR (ml/min/1.73 m^2^)	70.3 ± 21.8	56.3 ± 31.5	0.499
Smoking			
Present smoker	2 (9.1 %)	0 (0 %)	
Past smoker	7 (31.8 %)	8 (88.9 %)	0.015
Never	13 (59.1 %)	1 (11.1 %)	
Brinkman index	471.4 ± 726.8	553.3 ± 403.3	0.157
Coronary stenosis			
2-vessel	9 (40.9 %)	3 (33.3 %)	
1-vessel	6 (27.3 %)	2 (22.2 %)	0.801
0-vessel	7 (31.8 %)	4 (44.4 %)	
Prior PCI	4 (18.2 %)	1 (11.1 %)	0.627
β-blocker	7 (31.8 %)	6 (66.7 %)	0.074
Ca-blocker	11 (50.0 %)	5 (55.6 %)	0.779
ACE-inh or ARB	12 (54.5 %)	5 (55.6 %)	0.959
Symptom			
Typical chest pain	3 (13.6 %)	0 (0 %)	
Atypical chest pain	5 (22.7 %)	2 (22.2 %)	0.492
Asymptomatic	14 (63.6 %)	7 (77.8 %)	

Subjects with reduced HR response frequently had a smoking habit and tended to take β-blocker. *HRR* heart rate response, *eGFR* estimated glomerular filtration rate, *PCI* percutaneous coronary intervention, *ACE*-*Inh* angiotensin-converting enzyme inhibitor, *ARB* angiotensin receptor blocker

**Fig. 1:**
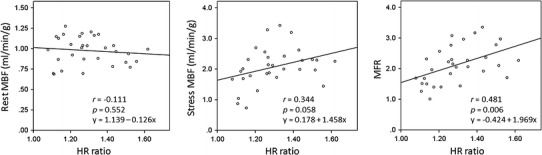
Linear regression analyses between heart rate (HR) ratio and quantitative values of remote regions. There was a good correlation between HR response to adenosine and myocardial flow reserve of remote regions

During the adenosine stress test, no patient had any significant adverse reactions, and required a reduction in the dose of adenosine or administration of aminophylline. Hemodynamic responses to adenosine, myocardial perfusion, and gated PET analyses between the groups are shown in Table 2. Average HR increased by 23.3 beats/min in the normal HR response group compared with 9.6 beats/min in the reduced HR response group. The subjects with reduced HR response had a significantly higher baseline HR than those with normal HR response (73.3 ± 10.4 vs. 64.7 ± 7.6, *p* = 0.041). Symptoms (chest pain or chest compression) during adenosine infusion were frequently observed in the normal HR response group (*p* = 0.036). There were no differences in BP and semi-quantitative myocardial perfusion (SRS, SSS, and SDS) between the groups. Stress MBF and MFR of remote regions (supplied by normal coronary arteries) were significantly lower in the reduced HR response group than those in the normal HR response group (stress MBF: 1.559 ± 0.517 vs. 2.279 ± 0.530, *p* = 0.004, MFR: 1.59 ± 0.36 vs. 2.35 ± 0.53, *p* = 0.001). However, there was no significant difference between the groups with respect to rest MBF (*p* = 0.794). Rate-pressure product tended to be higher in the reduced HR response group than that in the normal HR response group (9935 ± 3147 vs. 8336 ± 1583, *p* = 0.240). When rest MBF was modified by rate-pressure product, rest MBF and MFR in the reduced HR response group tended to be lower than that in the normal HR response group (*p* = 0.090 and 0.177, respectively). Rest and stress MBF of stenosed regions between the groups were statistically similar (rest MBF: 1.085 ± 0.245 vs. 0.988 ± 0.126, *p* = 0.458, stress MBF: 1.698 ± 0.362 vs. 2.077 ± 0.598, *p* = 0.239), while MFR of stenosed regions in the reduced HR response group was lower than that in the normal HR response group (1.59 ± 0.23 vs. 2.10 ± 0.50, *p* = 0.050). In addition, stress EF in the reduced HR response group was significantly lower than that in the normal HR response group (49.7 ± 9.6 vs. 60.3 ± 9.5, *p* = 0.003). Rest EF also tended to be lower in the reduced HR response group (55.7 ± 13.1 vs. 65.2 ± 8.9, *p* = 0.090). Linear regression analyses between HR response and quantitative values are shown in Fig. 1. A good correlation was observed between HR ratio and MFR of remote regions (*r* = 0.481).

**Table 2:** Hemodynamic responses to adenosine, myocardial perfusion, and gated PET analyses in normal and reduced heart rate response (HRR) groups

Variable	Normal HRR (n = 22)	Reduced HRR (n = 9)	*p* value
Rest HR (bpm)	64.7 ± 7.6	73.3 ± 10.4	0.041
Peak HR (bpm)	88.0 ± 10.0	82.9 ± 11.5	0.305
Heart rate ratio	1.37 ± 0.12	1.13 ± 0.03	<0.001
Rest mean BP (mmHg)	88.1 ± 11.2	94.9 ± 12.1	0.207
Minimum mean BP (mmHg)	79.4 ± 10.3	87.7 ± 15.3	0.151
ΔBP (minimum–rest) (mmHg)	−6.5 ± 8.3	−7.1 ± 8.4	0.617
Baseline rate-pressure product^a^	8336 ± 1583	9935 ± 3147	0.240
Symptoms during adenosine infusion	8 (36.4 %)	0 (0 %)	0.036
ST change during adenosine infusion	9 (40.9 %)	3 (33.3 %)	0.694
SRS	1.7 ± 3.0	2.6 ± 4.2	0.834
SSS	5.5 ± 7.3	7.9 ± 7.9	0.386
SDS	3.8 ± 5.6	5.3 ± 6.0	0.372
Rest MBF (ml/min/g)	0.980 ± 0.142	0.965 ± 0.215	0.794
Modified rest MBF (ml/min/g)^b^	1.060 ± 0.193	0.901 ± 0.219	0.090
Stress MBF (ml/min/g)	2.279 ± 0.530	1.559 ± 0.517	0.004
MFR	2.35 ± 0.53	1.59 ± 0.36	0.001
Modified MFR^c^	2.20 ± 0.53	1.83 ± 0.74	0.177
Rest EDV (ml)	100.6 ± 31.1	129.6 ± 56.4	0.164
Rest ESV (ml)	36.3 ± 18.0	64.1 ± 43.4	0.139
Rest EF (%)	65.2 ± 8.9	55.7 ± 13.1	0.090
Stress EDV (ml)	113.0 ± 36.4	138.4 ± 56.2	0.231
Stress ESV (ml)	47.0 ± 23.8	74.2 ± 42.4	0.074
Stress EF (%)	60.3 ± 9.5	49.7 ± 9.6	0.003
ΔEDV (stress–rest) (ml)	12.4 ± 12.8	8.8 ± 6.8	0.500
ΔESV (stress–rest) (ml)	10.7 ± 9.8	10.1 ± 9.3	0.896
ΔEF (stress–rest) (%)	−4.9 ± 5.2	−5.9 ± 6.1	0.777

*BP* blood pressure, *SRS* summed rest score, *SSS* summed stress score, *SDS* summed differential score, *MBF* myocardial blood flow, *MFR* myocardial flow reserve, *EDV* end-diastolic volume, *ESV* end-systolic volume, *EF* ejection fraction

^a^Rate-pressure product = HR × systolic BP

^b^Modified rest MBF = rest MBF × (8800/rate-pressure product)

^c^Modified MFR = stress MBF/modified rest MBF

In a multiple stepwise regression analysis, MFR of remote regions, gender, age, BMI, hypertension, diabetes mellitus, dyslipidemia, eGFR, Brinkman index, the number of coronary artery stenosis, symptom, HR ratio (as numerical values), ΔBP, symptoms and ST changes during adenosine infusion, rest EDV, rest ESV, and rest EF were taken into account. HR ratio (*p* = 0.003), dyslipidemia (*p* = 0.033), and Brinkman index (*p* = 0.045) were identified as predictors of the change in MFR of remote regions (Table 3).

**Table 3:** Multiple stepwise regression analysis with stepping method criteria of probability of F to enter <0.05 and to remove >0.10

Variable	Standardized coefficients (β)	*p* value
HR ratio	0.480	0.003
Dyslipidemia	−0.329	0.033
Brinkman index	−0.308	0.045

Independent variables (*p* < 0.05) are listed

Sensitivity, specificity, PPV, and NPV for myocardial ischemia in normal and reduced HR response groups are listed in Table 4. Diagnostic accuracy in reduced HR response group was worse than that in normal HR response group.

**Table 4:** Diagnostic ability of visual assessment in ^13^N-ammonia PET studies for myocardial ischemia in normal and reduced HR response groups (>50 % stenosis on CAG was considered as a gold standard)

	Sensitivity	Specificity	PPV	NPV
Normal HRR (n = 66 territories)	70.8 % (17/24)	92.9 % (39/42)	85.0 % (17/20)	84.8 % (39/46)
Reduced HRR (n = 27 territories)	62.5 % (5/8)	84.2 % (16/19)	62.5 % (5/8)	84.2 % (16/19)

*HRR* heart rate response, *PPV* positive predictive value, *NPV* negative predictive value

## Discussion

Adenosine induces maximal myocardial hyperemia immediately and has a short half-life (<10 s) [18]. Hemodynamic responses during adenosine infusion include a slight increase in HR and a slight decrease in systemic BP. Adenosine induces baroreceptor reflex response to hypotension and directly stimulates the sympathetic nervous system regardless of BP changes, increasing HR [19].

In the present study, 29.0 % (9/31) of all subjects demonstrated reduced HR response during adenosine stress conditions. Even normal coronary subjects (one normal volunteer and three chronic heart failure patients) showed reduced HR response, suggesting that reduced HR response was not only dependent on coronary stenosis. The subjects with reduced HR response in this study were fewer than those reported in previous studies [5, 6]. This is probably because a 2-route method was adopted in this study as the adenosine injection protocol. Using this protocol, adenosine infusion was performed more effectively because suspending adenosine infusion during administration of ^13^N-ammonia was not necessary.

The subjects with reduced HR response frequently had a smoking habit, the use of β-blocker, higher rest HR, lower stress MBF and MFR of remote regions, and lower EF in the present study. Smoking habit and the use of β-blocker might deteriorate the pharmaceutical action of adenosine. Higher rest HR and lower EF in the reduced HR response group were consistent with the findings of previous studies [4–6]. In these studies with comparatively larger populations on myocardial perfusion SPECT, reduced HR response was related to SRS and SSS, and not to SDS; however, the present preliminary study did not demonstrate differences of SRS, SSS, and SDS between the groups. Regardless of similar semi-quantitative perfusion scores, stress MBF and MFR of remote regions were lower in the reduced HR response group than those in the normal HR response group. It is considered that lower stress MBF and MFR of remote regions with reduced HR response were associated with inadequate reaction to adenosine infusion. When rest MBF was modified by rate-pressure product, rest MBF and MFR tended to be lower in the reduced HR response group than those in the normal HR response group. Rate-pressure product correction made differences of MFR between the groups smaller and less significant. The subjects with reduced HR response were supposed to have increased cardiac work and higher level of intrinsic adenosine at rest. Extrinsic adenosine response could be insufficient because intrinsic adenosine has already made coronary arteries dilated and coronary arteries did not have sufficient vasodilator capacity. Moreover, because stress MBF was significant lower in the reduced HR response group, non-responders whom adenosine did not have work well regardless of rate-pressure product were thought to truly exist. Therefore, reduced MFR could reflect different hemodynamics between subjects with normal and reduced HR response. For normal HR response subjects, reduced MFR represents coronary stenosis and/or microvascular and endothelial dysfunctions. On the other hand, reduced MFR means insufficient adenosine response in addition to the above (coronary stenosis and/or microvascular and endothelial dysfunctions) for reduced HR response subjects. Then, absolute (non-corrected) rest MBF and MFR were essential for accurate stratification of reduced MFR. Because stress MBF and MFR of stenosed regions were influenced by coronary stenosis in addition to adenosine response and microvascular and endothelial dysfunctions, quantitative values of remote regions were more useful when adenosine response was assessed. Differences of stress MBF and MFR between the groups were more evident in remote regions than those in stenosed regions.

In addition, the subjects with reduced HR response did not have symptoms during adenosine infusion in this study. That is, non-responders to adenosine had lower HR response and lesser symptoms during adenosine infusion, and lower MFR of remote regions. Moreover, a good correlation between HR response and MFR was indicated by linear regression analysis. To our knowledge, the present study was the first to investigate the association between reduced HR response to adenosine and quantitative values on ^13^N-ammonia PET studies. Further studies measuring blood caffeine levels are desired. A part of the cause of inadequate response to adenosine can be revealed by measuring blood caffeine levels.

MFR of remote regions is changed by microvascular and vascular endothelial dysfunctions besides the reaction to adenosine [10–13]. A multiple stepwise regression analysis revealed that HR response to adenosine was an independent predictor of the change in MFR of remote regions, similarly to dyslipidemia and smoking (microvascular and vascular endothelial dysfunctions).

It has previously been reported that the ability to detect coronary artery disease and the diagnostic accuracy of adenosine myocardial perfusion imaging are not affected by variations of hemodynamic response [20, 21]. However, because patients with reduced HR response have lower MFR of remote regions, dilatation of coronary artery was certainly also diminished, leading to underestimation of scar or ischemia. In the present study, diagnostic accuracy of visual assessment for myocardial ischemia in the reduced HR response group was lower than that in the normal HR response group. Low sensitivity in the reduced HR response group was probably due to inadequate effectiveness of adenosine infusion. This deteriorated pharmaceutical action also might yield inhomogeneous myocardial accumulation under stress condition, resulting in reduced specificity. Further studies including larger population and setting functional flow reserve (FFR) as a gold standard would be necessary for validation.

## Conclusions

The subjects with reduced HR response to adenosine infusion had lower stress MBF and MFR of remote regions and lower EF. HR response and MFR of remote regions correlated well, and HR response was one of the predictors of the change in MFR. Therefore, careful consideration should be taken into for visual and quantitative analyses of these patients.
